# Socio-Demographic and Clinical Characteristics of First-Ever Admitted Psychiatric Inpatients in Palestine: A Cross-Sectional Study

**DOI:** 10.1192/bjo.2024.202

**Published:** 2024-08-01

**Authors:** Nizar Marzouqa, Thaer Alhroob, Wala Alnammourah, Layth Oweina, Asala Awwad

**Affiliations:** Bethlehem Psychiatric Hospital, Bethlehem, Palestine

## Abstract

**Aims:**

Bethlehem Psychiatric Hospital is the only psychiatric hospital in the West Bank. Epidemiological data on mental health in Palestine is very limited due to the lack of research in this area. This study aims to evaluate the socio-demographic characteristics and clinical outcomes of first-time admitted patients at Bethlehem Psychiatric Hospital over a year period.

**Methods:**

A retrospective cross-sectional study was conducted at the Bethlehem Psychiatric Hospital, reviewing the medical records of patients admitted for the first time between October 2022 and October 2023. Data collection was conducted manually by residents through an Excel sheet. Next, demographic characteristics (socioeconomic and demographic measures), relevant history (past medical, psychiatric, and forensic), presenting episode characteristics, and current hospitalization (admission, hospital stay, and discharge) were studied descriptively.

**Results:**

Of the 140 patients admitted for the first time to the psychiatric hospital between October 2022 and October 2023, the mean age was 32.6, a majority (70%) were male, more than half were single, around two-thirds finished high school, and 13.6% had a university degree. Only 41.4% of patients were referred from a medical or legal source. The overwhelming majority of the patients had poor prior outpatient follow-up.

Substance use was common (Tobacco: 63.8%, alcohol: 16.1%, and other substances: 26.4%). Family history of psychiatric illness was found in 40% of the cases, and prior imprisonment in 42%.

The most common presenting complaints included: sleep disturbances (84.3%), and physical aggression (73.6%). Delusions were elicited in 72.9% (most commonly persecutory-paranoid, and least commonly grandiose and reference delusions). Hallucinations were present in 38.6% of admissions, auditory hallucinations were the most common. 15.4% had depressed mood, and 22.1% had current suicidal ideations.

Involuntary admissions constituted 62.1% of all admissions. A third of urine drug tests, conducted in 68.6%, came back positive. Patients stayed a mean of 17.8 days. A diagnosis was reached in 82.1% of patients, the most common established diagnosis was Schizophrenia spectrum (42.9%). Around a third were discharged against medical advice.

**Conclusion:**

This is the first study conducted on Palestinian psychiatric inpatients. The results of this study suggest that most patients who are admitted had poor outpatient care. Delusions were elicited in the majority of patients, amongst different final diagnoses.

There is a need for more research on Palestinian psychiatry, integrative social services, and better mental health regulations to protect the rights of mental health patients.

